# Repeated Detection of Rubella Virus IgM Antibodies in Two Pregnancies Without Evidence of Fetal Infection: A Case Report and Challenges in Serological Interpretation

**DOI:** 10.7759/cureus.86002

**Published:** 2025-06-14

**Authors:** Faris Kazic, Bedrana Muracevic-Begovic, Enid Nakicevic

**Affiliations:** 1 Obstetrics and Gynecology, Cantonal Hospital Zenica, Zenica, BIH

**Keywords:** false positive, igm, pregnancy, rubella virus, torch

## Abstract

Serologic screening for rubella virus, as part of TORCH testing during pregnancy, remains a cornerstone in the prevention of congenital rubella syndrome. However, isolated detection of rubella-specific IgM antibodies may lead to diagnostic uncertainty, especially in the absence of clinical signs or supporting serological evidence. We present a case of a pregnant woman who tested positive for rubella IgM antibodies during two consecutive pregnancies, despite lacking any sonographic or serologic indications of fetal infection. Both pregnancies culminated in the delivery of healthy neonates with no clinical or laboratory evidence of congenital rubella. This case highlights the possibility of false-positive IgM results, persistent IgM antibodies, or nonspecific immune reactivity. Timely clarification of such findings is essential to avoid unnecessary anxiety and invasive procedures. Interpretation of rubella IgM results should always consider the clinical context, supplemental serologic tests (such as IgG avidity), and current epidemiological data.

## Introduction

Rubella is a mild viral illness; however, infection during pregnancy can lead to serious consequences, including congenital rubella syndrome (CRS). In pregnant women, rubella may be asymptomatic or present with symptoms such as upper respiratory tract involvement, fever, lymphadenopathy, especially suboccipital and retroauricular, and a maculopapular rash [1,2].

In fetuses of infected mothers, a wide spectrum of outcomes can occur, ranging from multiple congenital anomalies collectively referred to as congenital rubella syndrome to fetal death. The most common manifestations of CRS include intrauterine growth restrictions, microcephaly, meningoencephalitis, cataracts, hearing impairment, cardiac defects, and radiolucent bone disease [3,4,5]. 

Rubella-specific IgM antibodies are used as a marker of acute infection; however, their interpretation can be challenging due to possible persistence or nonspecific reactivity. The immune response to natural rubella infection typically involves the early appearance of IgM antibodies, which can persist for several weeks to months, followed by the development of IgG antibodies that confer long-lasting immunity. By contrast, rubella vaccination induces a similar antibody response, but IgM antibodies generated post-vaccination are generally of lower intensity and shorter duration. Importantly, the presence of IgM antibodies after vaccination does not necessarily indicate an active or recent infection but rather reflects the expected immune reaction to the vaccine antigen. Recognizing these differences is critical in the clinical interpretation of serological results during pregnancy, as it helps differentiate between true acute infection and post-vaccinal antibody responses. [6,7].

We present a case of a woman with repeated positive rubella IgM findings in two consecutive pregnancies, without any confirmation or fetal infection. 

## Case presentation

The patient is a 21-year-old healthy woman in her second pregnancy. At 24 weeks of gestation, a routine gynecological examination revealed dilation of the posterior horn of the lateral cerebral ventricle measuring 9.7 mm. TORCH testing was recommended. TORCH test is a serological panel used to detect maternal antibodies against a group of infectious agents known to cause congenital infections and fetal complications during pregnancy. The test typically involves enzyme-linked immunosorbent assays (ELISA) or chemiluminescent immunoassays (CLIA) to measure specific IgM and IgG antibodies in maternal or fetal serum. The results of TORCH serological screening at 24 weeks of gestation are presented in Table 1.

**Table 1:** Results of TORCH serological screening at 24 weeks of gestation TORCH: an acronym that refers to a group of perinatal infections that can cause congenital anomalies if transmitted from the mother to the fetus. The term stands for T - *Toxoplasma gondii*, O - other infections (*HIV*, *Hepatitis B*, *Varicella Zoster virus*), R -* Rubella virus*, C - *Cytomegalovirus*, and H - *Herpes simplex virus* (types 1 and 2). UI/ml - International units per mililiter The presence of CMV IgG positivity with CMV IgG negativity typically indicates past exposure to cytomegalovirus and the development of long-term immunity.

Analysis	Referent range	Result
Toxoplasma gondii IgM	Negative < 0.55 UI/ml	0.19 UI/ml (Negative)
	Retest > 0.55 and < 0.65 IU/ml	
	Positive > 0.65 IU/ml	
Toxoplasma gondii IgG	Negative < 4 IU/ml	0 UI/ml (Negative)
	Retest > 4 and < 8 IU/ml	
	Positive > 8 IU/ml	
Cytomegalovirus IgM	Negative < 0.70 IU/ml	0.05 UI/ml (Negative)
	Retest > 0.70 and < 0.90 IU/ml	
	Positive > 0.90 IU/ml	
Cytomegalovirus IgG	Negative < 4 IU/ml	15.80 UI/ml (Positive)
	Retest > 4 and < 6 IU/ml	
	Positive > 6 IU/ml	
Rubella virus IgM	Negative < 0.80 IU/ml	1.87 UI/ml (Positive)
	Retest > 0.80 and < 1.20 IU/ml	
	Positive > 1.20 IU/ml	
Rubella virus IgG	Negative < 10 IU/ml	89.30 UI/ml (Positive)
	Retest > 10 and < 15 IU/ml	
	Positive > 15 IU/ml	
Herpes simplex virus IgM	Negative < 0.90 IU/ml	0.237 UI/ml (Negative)
	Retest > 0.90 and < 1.1 UI/ml	
	Positive > 1.1 UI/ml	
Herpes simplex virus IgG	Negative < 0.90 IU/ml	4.474 UI/ml (Positive)
	Retest > 0.90 and < 1.1 UI/ml	
	Positive > 1.1 UI/ml	

An anomaly scan was subsequently performed and showed normal findings. The width of the bilateral choroid plexus was within normal limits, and fetal biometry was appropriate for gestational age, anterior placenta, and normal amniotic fluid volume. Due to suspicion of a false-positive result, TORCH testing was repeated 14 days later. Although additional testing, such as IgG avidity or PCR for rubella, would have been helpful in distinguishing between acute and past infection, these assays were not routinely available in our laboratory setting at the time of evaluation. In Bosnia and Herzegovina, both IgG avidity and PCR testing for rubella are not part of standard diagnostic protocols and are generally limited to specialized or reference laboratories, often requiring sample referral abroad. Given these constraints, we relied on serological follow-up and the clinical context, with repeat testing conducted precisely 14 days later, which aligns with local diagnostic capabilities. The results of TORCH serological screening at 26 weeks of gestation are presented in Table 2.

**Table 2:** TORCH serological screening results at 26 weeks of gestation TORCH: an acronym that refers to a group of perinatal infections that can cause congenital anomalies if transmitted from the mother to the fetus. The term stands for T - Toxoplasma gondii, O - other infections (HIV, Hepatitis B, Varicella Zoster virus), R - Rubella virus, C - Cytomegalovirus, and H - Herpes simplex virus (types 1 and 2). UI/ml- International units per mililiter The presence of CMV IgG positivity with CMV IgG negativity typically indicates past exposure to cytomegalovirus and the development of long-term immunity.

Analysis	Referent range	Result
Toxoplasma gondii IgM	Negative < 0.55 UI/ml	0.02 UI/ml (Negative)
	Retest > 0.55 and < 0.65 IU/ml	
	Positive > 0.65 IU/ml	
Toxoplasma gondii IgG	Negative < 4 IU/ml	0 UI/ml (Negative)
	Retest > 4 and < 8 IU/ml	
	Positive > 8 IU/ml	
Cytomegalovirus IgM	Negative < 0.70 IU/ml	0.05 UI/ml (Negative)
	Retest > 0.70 and < 0.90 IU/ml	
	Positive > 0.90 IU/ml	
Cytomegalovirus IgG	Negative < 4 IU/ml	16 UI/ml (Positive)
	Retest > 4 and < 6 IU/ml	
	Positive > 6 IU/ml	
Rubella virus IgM	Negative < 0.80 IU/ml	3.50 UI/ml (Positive)
	Retest > 0.80 and < 1.20 IU/ml	
	Positive > 1.20 IU/ml	
Rubella virus IgG	Negative < 10 IU/ml	68 UI/ml (Positive)
	Retest > 10 and < 15 IU/ml	
	Positive > 15 IU/ml	
Herpes simplex virus IgM	Negative < 0.90 IU/ml	0.235 UI/ml (Negative)
	Retest > 0.90 and < 1.1 UI/ml	
	Positive > 1.1 UI/ml	
Herpes simplex virus IgG	Negative < 0.90 IU/ml	4.471 UI/ml (Positive)
	Retest > 0.90 and < 1.1 UI/ml	
	Positive > 1.1 UI/ml	

In her first pregnancy, three years earlier, similar findings were noted: positive rubella IgM and IgG and high cytomegalovirus levels, without any evidence of fetal infection. That pregnancy was monitored regularly without antiviral treatment. Delivery was by cesarean section due to fetal asphyxia; the newborn was clinically healthy, and postnatal rubella screening was negative. It should be noted that the first child was delivered when the mother was 17 years old, she had previously been vaccinated, and the pregnancy was uneventful. The findings at that time were interpreted as a delayed post-vaccination immune response. In Bosnia and Herzegovina, rubella vaccination is administered as part of the MMR vaccine, with the first dose typically given at 12 months of age and a second dose at six years, according to the national immunization schedule. However, in this particular case, the patient received her MMR revaccination at the age of 16 due to delayed vaccine availability and catch-up immunization efforts during that period. The vaccine shortage did not persist for an extended period but occurred shortly after the time when the patient was originally scheduled to receive her second dose. In addition, parental non-compliance contributed to the delay, resulting in the administration of the revaccination much later than recommended. Given the relatively short interval, approximately one year, between the revaccination and the onset of pregnancy, the possibility of persistent IgM antibodies as a post-vaccination response cannot be entirely excluded.

In the current pregnancy, after consultation with an infectious disease specialist and additional testing in a reference laboratory, the findings were most likely attributed to persistent IgM antibodies, false-positive reactivity (possibly due to cross-reactivity with other viruses or antibody interference), or an unspecific immune response

The pregnancy was completed at 39 weeks by cesarean section, resulting in the birth of a healthy neonate. TORCH screening of the newborn was conducted two days postpartum. The results of TORCH serological screening of the newborn are presented in Table 3.

**Table 3:** TORCH serological screening of the newborn TORCH: an acronym that refers to a group of perinatal infections that can cause congenital anomalies if transmitted from the mother to the fetus. The term stands for T - Toxoplasma gondii, O - other infections (HIV, Hepatitis B, Varicella Zoster virus), R - Rubella virus, C - Cytomegalovirus, and H - Herpes simplex virus (types 1 and 2). UI/ml - International units per mililiter

Analysis	Referent range	Result
Toxoplasma gondii IgM	Negative < 0.55 UI/ml	0.10 UI/ml (Negative)
	Retest > 0.55 and < 0.65 IU/ml	
	Positive > 0.65 IU/ml	
Toxoplasma gondii IgG	Negative < 4 IU/ml	0 UI/ml (Negative)
	Retest > 4 and < 8 IU/ml	
	Positive > 8 IU/ml	
Cytomegalovirus IgM	Negative < 0.70 IU/ml	0.01 UI/ml (Negative)
	Retest > 0.70 and < 0.90 IU/ml	
	Positive > 0.90 IU/ml	
Cytomegalovirus IgG	Negative < 4 IU/ml	17 UI/ml (Positive)
	Retest > 4 and < 6 IU/ml	
	Positive > 6 IU/ml	
Rubella virus IgM	Negative < 0.80 IU/ml	0.10 UI/ml (Negative)
	Retest > 0.80 and < 1.20 IU/ml	
	Positive > 1.20 IU/ml	
Rubella virus IgG	Negative < 10 IU/ml	107 UI/ml (Positive)
	Retest > 10 and < 15 IU/ml	
	Positive > 15 IU/ml	
Herpes simplex virus IgM	Negative < 0.90 IU/ml	0.044 UI/ml (Negative)
	Retest > 0.90 and < 1.1 UI/ml	
	Positive > 1.1 UI/ml	
Herpes simplex virus IgG	Negative < 0.90 IU/ml	6.518 UI/ml (Positive)
	Retest > 0.90 and < 1.1 UI/ml	
	Positive > 1.1 UI/ml	

No invasive testing was indicated during pregnancy. A simplified TORCH serology overview and newborn outcomes between the two pregnancies are presented in Table 4.

**Table 4:** Simplified TORCH serology overview and newborn outcomes between two pregnancies For the first pregnancy, only partial TORCH serology results were available. Rubella IgM and IgG were documented as positive, but numerical values and full TORCH panel data were not preserved in the medical archive.

Parameter	First pregnancy	Second pregnancy
Rubella IgM	Positive	Positive
Rubella IgG	Positive	Positive
Toxoplasma gondii IgM	Not available	Negative
Toxoplasma gondii IgG	Not available	Negative
Cytomegalovirus IgM	Not available	Negative
Cytomegalovirus IgG	Not available	Positive
Herpes simplex virus IgM	Not available	Negative
Herpes simplex virus IgG	Not available	Positive
Avidity testing	Not performed	Not performed
PCR	Not performed	Not performed
Neonatal outcome	Healthy newborn	Healthy newborn

## Discussion

Serological screening for TORCH infections, especially during pregnancy and the early postnatal period, represents an essential diagnostic tool for identifying potentially teratogenic infections [8]. However, interpretation of results, particularly rubella IgM, requires careful and contextual analysis to avoid incorrect clinical decisions [9]. In the presented case, a newborn was tested for rubella as part of the TORCH panel two days after birth, with results showing negative rubella IgM, positive rubella IgG, and positive cytomegalovirus IgG. In this case, the patient was CMV IgG positive and CMV IgM negative during pregnancy, with no clinical signs or ultrasound findings suggestive of active infection. The CMV IgG positivity was interpreted as a marker of past exposure and consistent with established maternal immunity.

Rubella IgM antibodies typically appear shortly after primary infection, but may persist after primary infection for several months, especially following vaccination, reducing the specificity of the test [10]. Furthermore, IgM test reactivity can be affected by other viral infections such as parvovirus B19, cytomegalovirus, or Epstein-Barr virus or due to technical assay interference [11]. In addition, high rubella IgG titers, especially when accompanied by high avidity, are indicative of past infection or immunization and effectively rule out recent infection during pregnancy [12]. The presence of strongly positive rubella IgG in a clinically unremarkable pregnancy and healthy newborn further supports the absence of congenital infection and negates the need for invasive testing.

Congenital rubella infection typically presents with a well-defined triad in the neonate, including cataracts, deafness, and congenital heart defects, often with intrauterine growth restriction [13,14]. In the absence of these clinical findings and with normal neonatal examination results, the likelihood of active infection is minimal. Therefore, PCR testing for rubella in amniotic fluid or neonatal blood was not indicated, given the absence of clinical suspicion. 

This case highlights the importance of integrating clinical and laboratory findings to avoid misinterpretation of serological results. Overreliance on isolated serological parameters, without consideration of clinical context and epidemiological background, may lead to unnecessary invasive procedures, increased parental anxiety, and additional burden on the healthcare system. Rational application of the TORCH panel, particularly in the third trimester or postnatal period, requires a thorough understanding of antibody kinetics, timing of infection, and potential laboratory interferences. In Bosnia and Herzegovina, TORCH screening is not a routine procedure performed in every pregnancy. It is usually indicated only when abnormalities are detected during ultrasound examinations or other clinical findings raise suspicion of infection. Given the possibility of false-positive rubella IgM results, confirmatory tests such as IgG avidity assays or PCR can be useful for more accurate infection dating.

Ultimately, a multidisciplinary approach involving consultation with infectious disease specialists, neonatologists, and laboratory physicians is essential for optimal decision-making. Education of healthcare providers on the limitations and interpretation of TORCH serology is critical for preventing overdiagnosis and ensuring evidence-based perinatal care.

## Conclusions

This case highlights the importance of cautious interpretation of rubella IgM results during pregnancy. Routine serologic screening can lead to false-positive findings, posing a risk of unnecessary stress, additional testing, and potentially invasive procedures. Reviewing maternal vaccination history is crucial in the serological interpretation of infectious disease markers during pregnancy. Vaccination can induce antibody responses, including IgM and IgG production, that may mimic those seen in natural infections. Understanding a patient's vaccination status helps differentiate between vaccine-induced antibodies and those resulting from active or recent infection. Furthermore, incorporating IgG avidity testing and consulting with infectious disease specialists can support better-informed clinical decision-making.

In addition, this case underscores the need for clinicians to understand antibody dynamics during pregnancy and the postpartum period. Serological results must not be interpreted in isolation, but rather within the context of comprehensive clinical evaluation. False-positive IgM results may occur due to cross-reactivity and assay limitations. The combination of normal perinatal outcomes, absence of neonatal symptoms, and reassuring serology supports the exclusion of congenital infection. Careful case-by-case assessment remains essential to avoid misdiagnosis and ensure appropriate clinical management.
